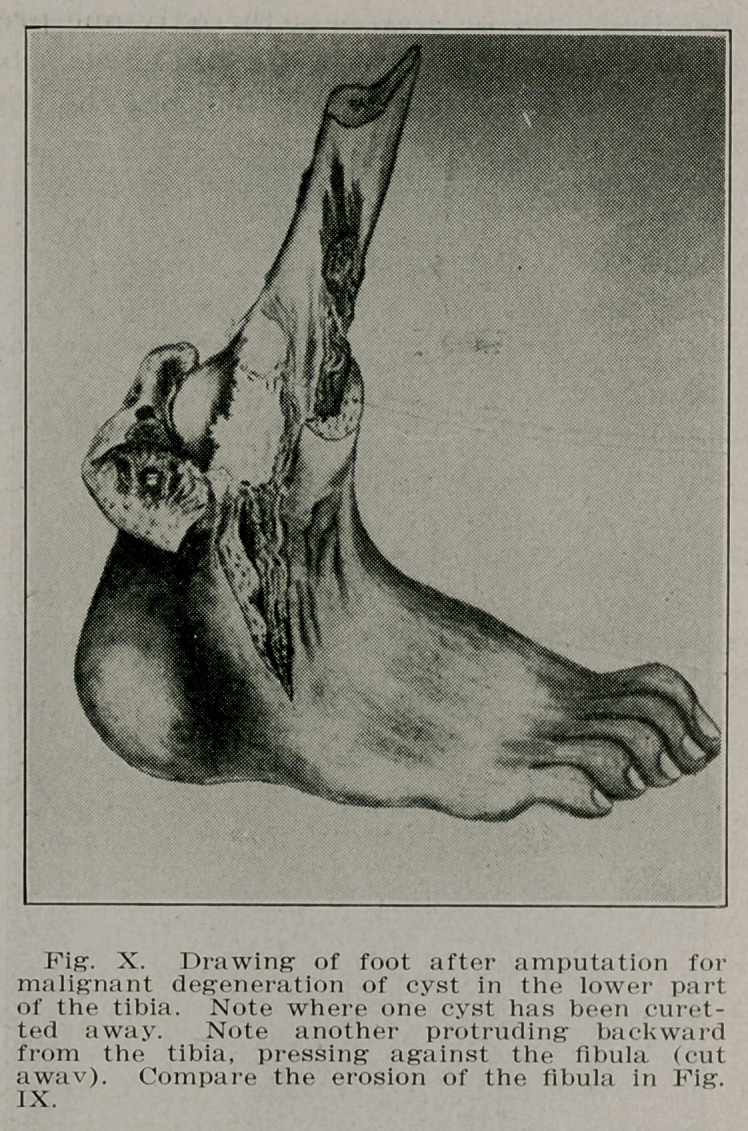# Cysts of Bone

**Published:** 1914-05

**Authors:** A. F. Tyler

**Affiliations:** Omaha, Neb.


					﻿CYSTS OF BONE
A. F. Tyler, M. D., Omaha, Neb.
Many formerly held that cysts of bone were part of a degen-
erative process taking place in osteomata, chondromata and
sarcomata osteomalacia and osteitis deformans. Indeed in
the American Text-book of Pathology of a few years ago the
statement is made that cysts of bone seldom occur from any
other cause. The ability to find these cysts in the living sub-
ject by means of the X-ray has shown that they are much more
frequent than was formerly supposed and that cysts of bone
do exist in no way connected with any other kind of bone
pathology. Simple cysts are found in the long bones quite
often. Cysts of the lower jaw, where they form about an un-
erupted tooth, or about the root of an old useless tooth, are
piost frequent of all. Those cases of multiple cysts in bone are
thought to be part of a chronic inflammatory process where
the bone softens in a manner similar to osteoralacia.
It is in this latter class that spontaneous fractures occur.
The portion of the bone involved becomes porous and very
fragile being filled with many small cysts. These cyst cavities
have a distinct lining membrane or sac (Fig. Ill) and are filled
with a viscid substance similar to the white of egg.
The outlook for recovery in simple cysts is good under
proper treatment. In the multiple type, the condition grad-
ually extends unless the bone is resected. Simple curretting
the cysts will not stop the process but resection with bone
graft to fill the gap results in good function. The simple.type
can be curetted thoroughly so as to destroy the lining mem-
brane, then the cavity is painted with pure carbolic acid fol-
lowed by alchol and is packed with iodoform gauze. At the
end of forty-eight hours the packing is removed and the. cavity
filled with Moerhof’s bone wax:
Iodoform ............................ 60
Spermaceti .......................... 40
Oil of sesame........................ 40
Case I.—W. M., a school boy of 1'2 was playing ball when
his “left foot caught behind the right” and he could not get
up. A physician was called and treated the fracture of the
left femur just below the trochanter. Recovery was unevent-
ful but four months later the boy turned quickly in the house
and fell to the floor. The same physician was called and
found a fracture of the same femur at the same place as be-
fore. He brought the boy to St. Joseph’s Hospital where a
radiograph revealed a polycystic condition of the left femur
involving the bone from the surgical neck to well below the
trochanter, a fracture was also present extending transversely
across the shaft just below the trochanters (see Fig. 1). A
diagnosis of osteitis fibrosa cyseiea was made and operation
recommended. The cysts were curetted, the bone was resected
and a graft from the crest of the tibia was inserted from the
two fragments extending into the medulla of each fragment.
The patient was able to leave the hospital in four months
(Figs. I, 11 and HI).
Case II.—Mrs. E. B., 35, a housewife and mother doing her
own work came complaining that at times her left ankle be-
came painful and swollen. A few days rest restored it to nor-
mal function. Radiographs revealed a cyst cavity in the lower
end of the tibia within one inch of the articular surface and
with oidy a shell of bone covering the anterior surface. In-
cision and curettement removed a viscid substance from the
cavity leaving healthy bone. The cavity was packed with
iodoform gauze for forty-eight hours. When this was removed
the cavity was filled with Moerhof’s bone wax. Recovery was
complete and the patient has full function today (Figs. IV
and V).
Case III.—F. R., a school boy of 10 rolled off his sled last
February fracturing the right femur at the junction of the
middle and lower third. Radiographs made to show the apposi-
tion revealed a cyst lying in the shaft of the bone just below
the site of rfacture. Radiographs were then made of the en-
tire body but this solitary cyst was the only one present. The
fracture united so that later radiographs failed to show its
presence. The cyst remained unoperated, but the boy has per-
fect function. Radiographs made September 11, 1913, reveal
a second cyst forming in the femur below the first and a third
in the fibula of the same leg (Figs. VI, VII and VIII).
Case V.—C. II., a farmer of 57 came complaining of a pain-
ful swelling near the ankle on the left leg. Examination re-
vealed a solid swelling near the ankle of the left leg. Radio-
graphs showed a large cyst cavity in the tibia and an erosion
of the fibula at the same level. The posterior tibial artery was
calcareous, showing plainly in the radiograph, and was pushed
backward at the level of the cyst. A diagnosis was made of
a large cyst which had ruptured through the bone into the
soft tissues and by direct pressure had displaced the artery.
Operation showed this to be the condition, the cavity being
more than filled by a jelly-like substance. Microscopical ex-
amination of the curettement from the walls of the cavity
showed a small round cell infiltration and on the strength of
this the leg was amputated, the thickening being considered
as having undergone carcinomatous degeneration (see Figs.
IX and X).
				

## Figures and Tables

**Fig. I. f1:**
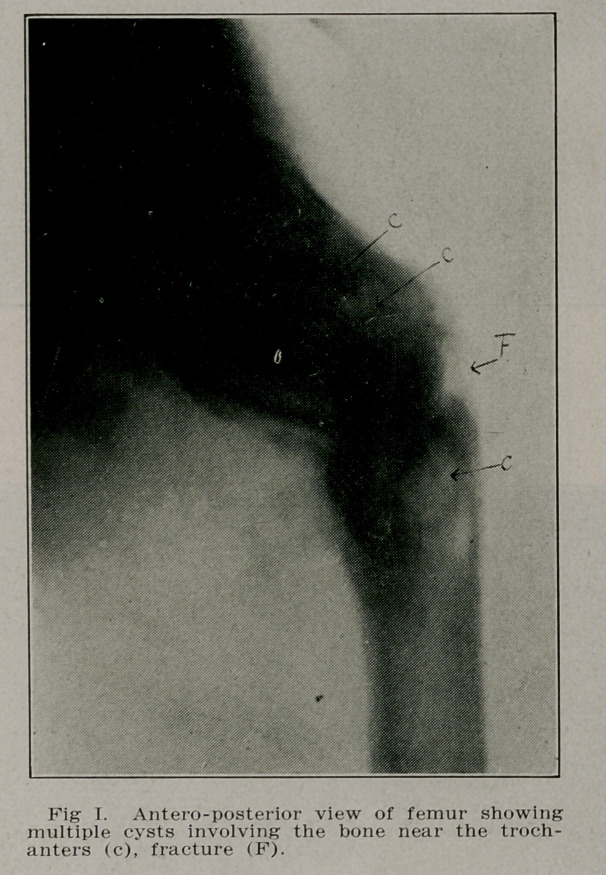


**Fig. II. f2:**
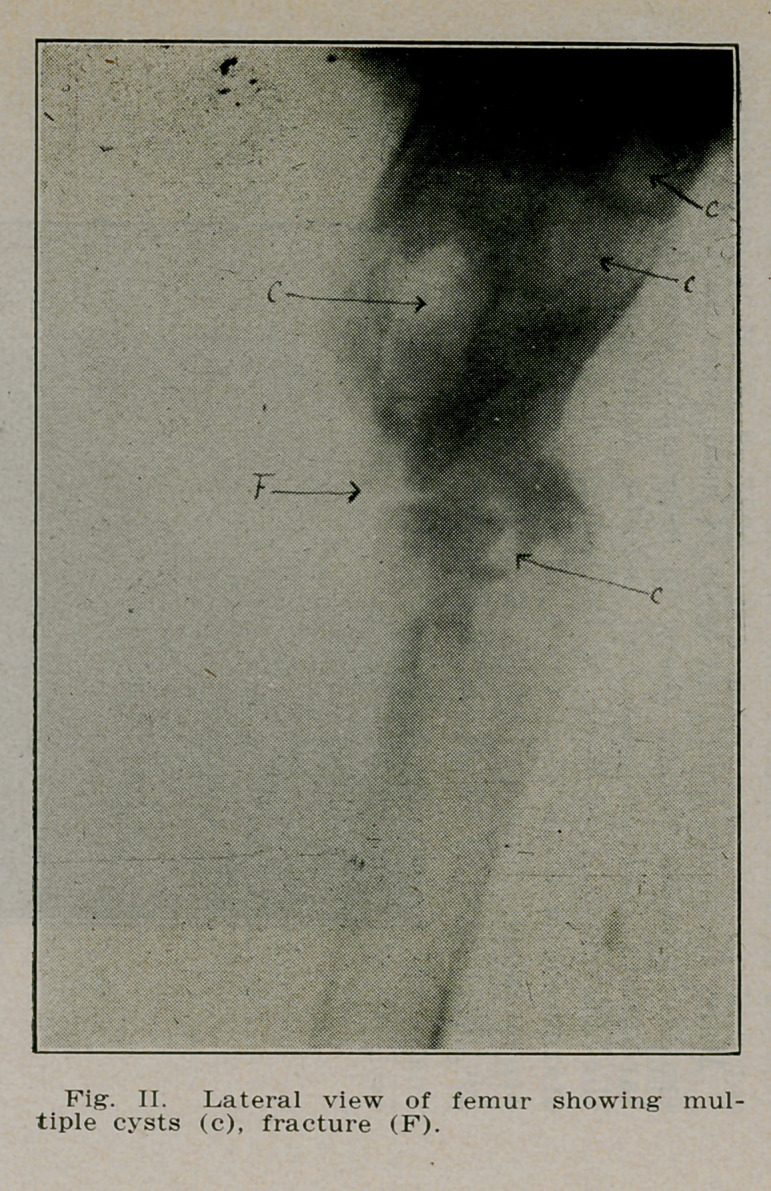


**Fig. III. f3:**
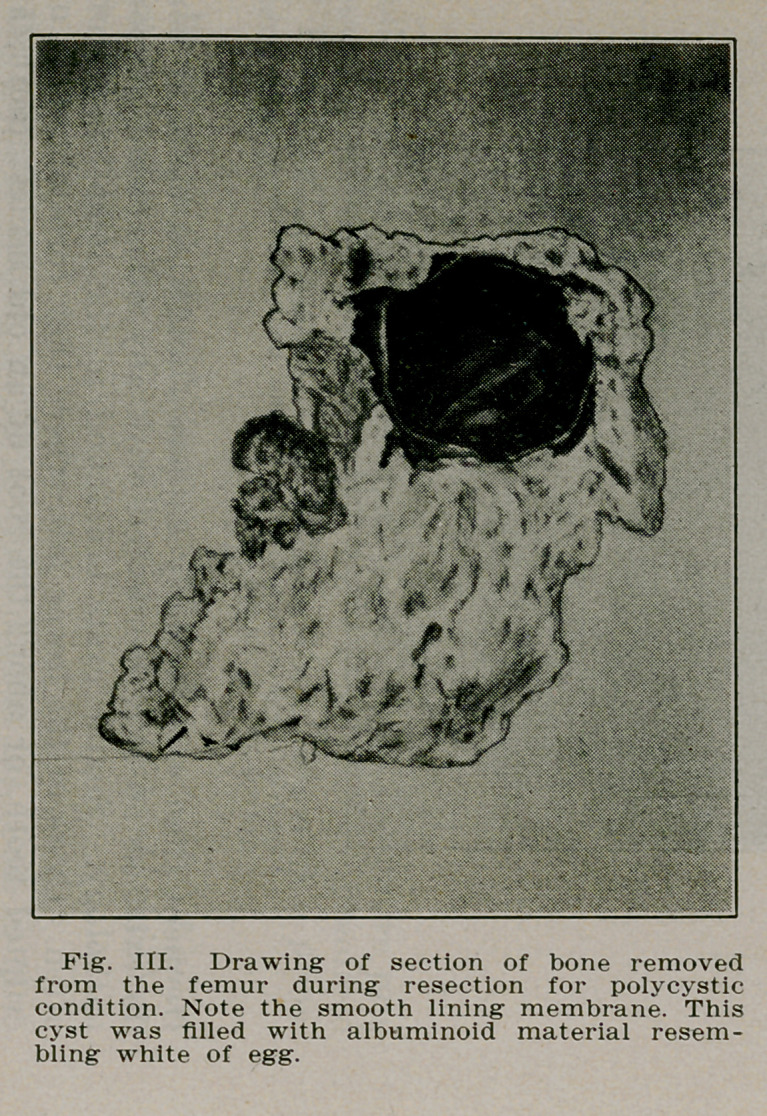


**Fig. IV. f4:**
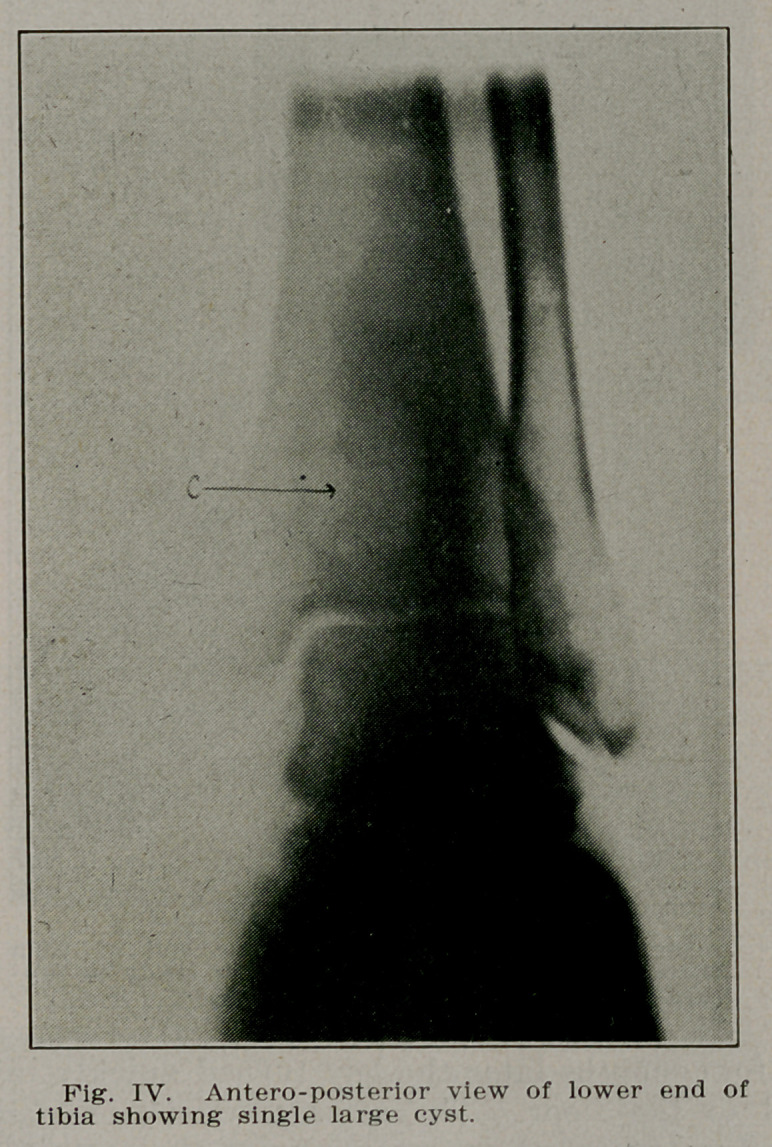


**Fig. V. f5:**
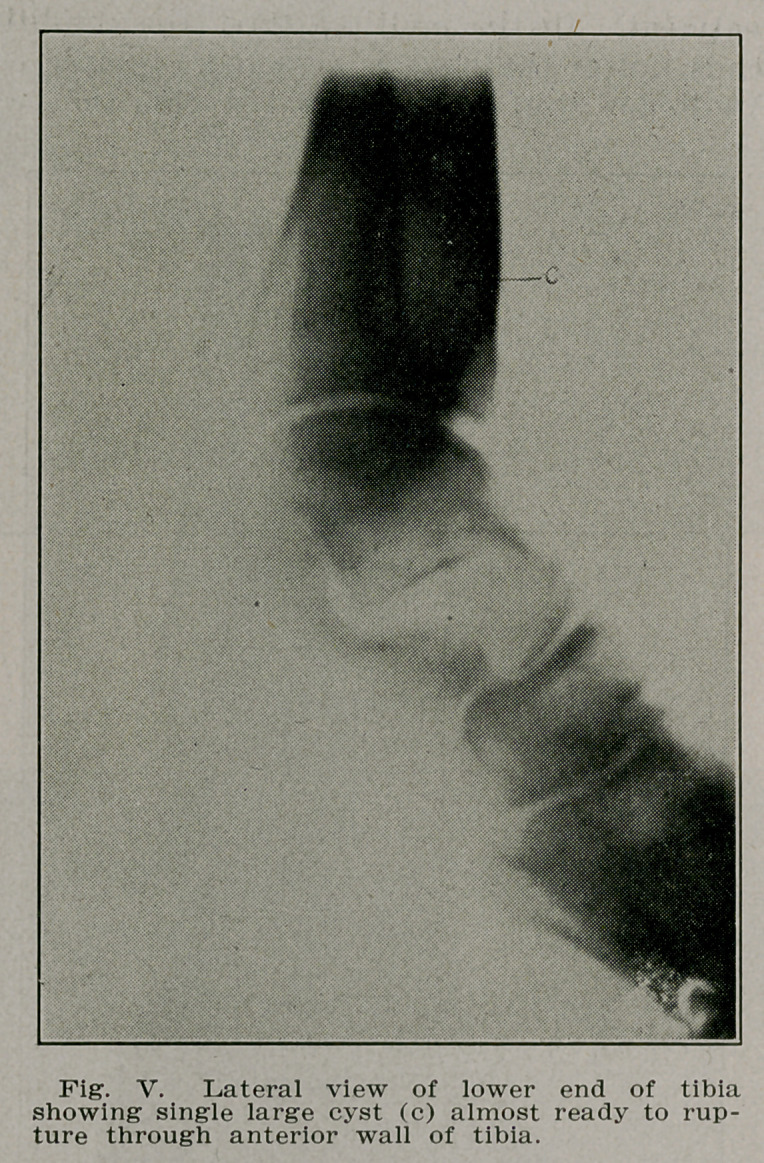


**Fig. VI. f6:**
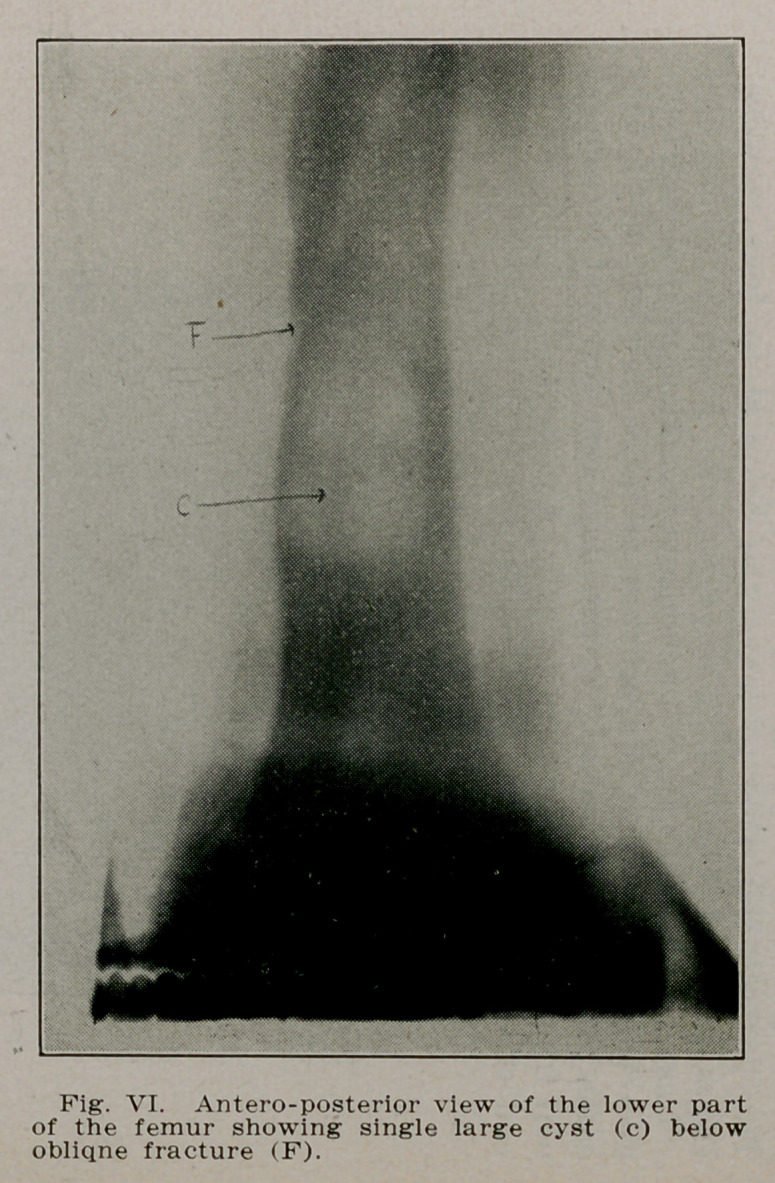


**Fig. VII. f7:**
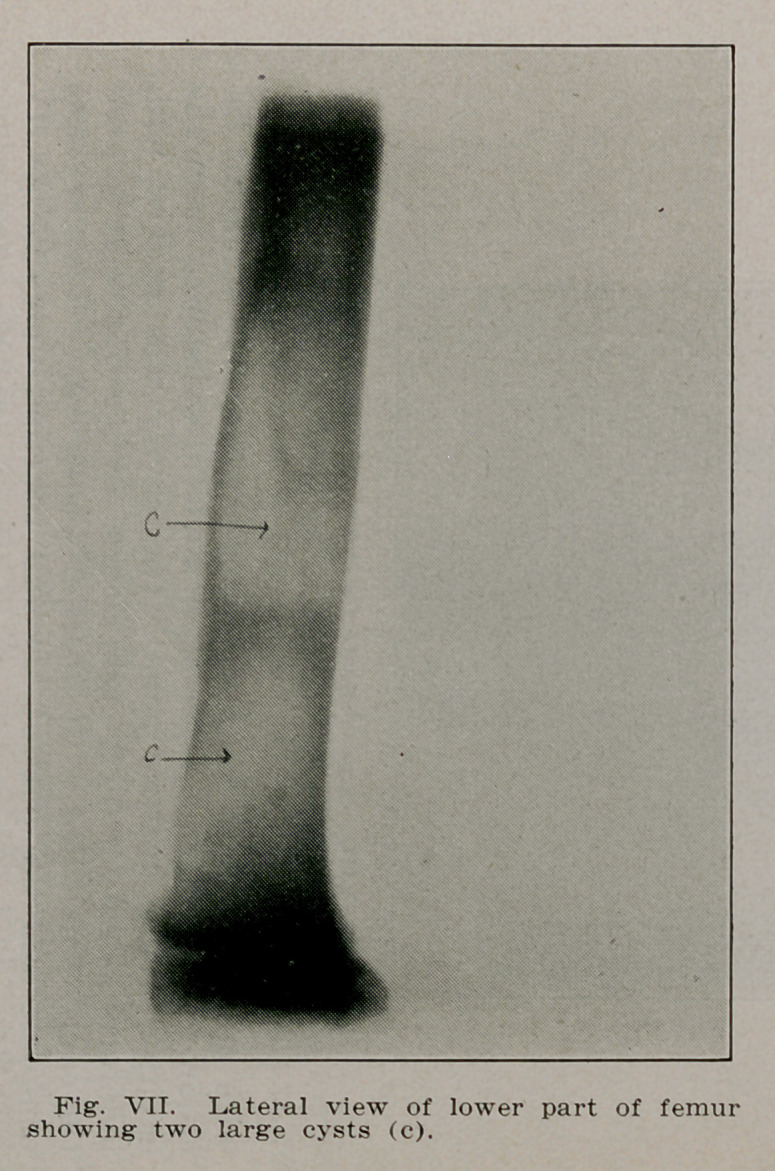


**Fig. VIII. f8:**
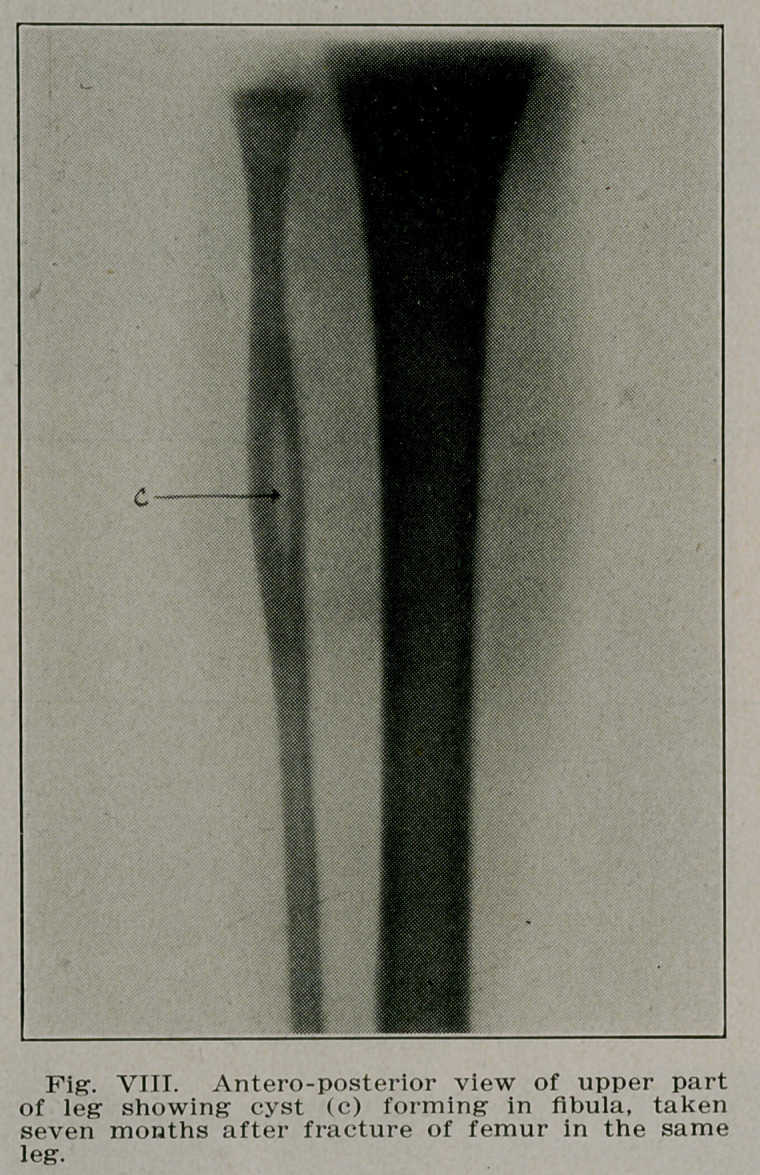


**Fig. IX. f9:**
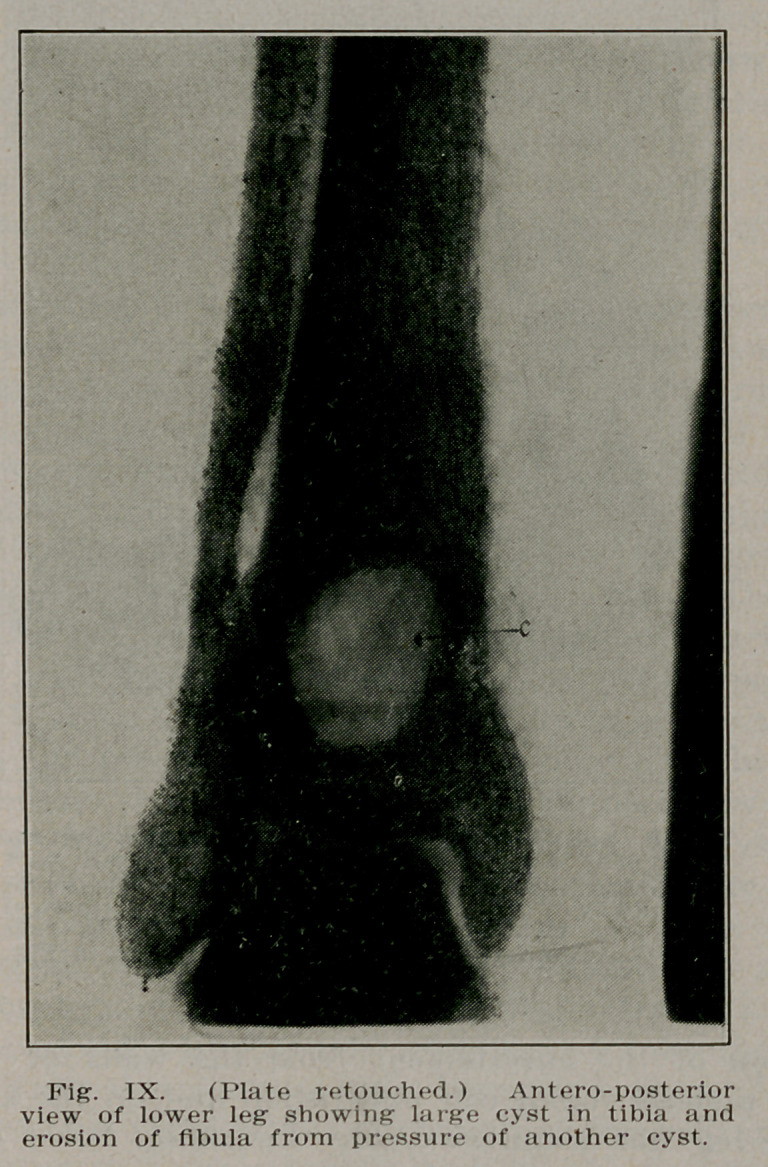


**Fig. X. f10:**